# A Target-Site Mutation Confers Cross-Resistance to ALS-Inhibiting Herbicides in *Erigeron sumatrensis* from Brazil

**DOI:** 10.3390/plants11040467

**Published:** 2022-02-09

**Authors:** Vanessa Vital Silva, Rafael Mendes, Andreia Suzukawa, Fernando Adegas, Francismar Marcelino-Guimaraes, Rubem Oliveira

**Affiliations:** 1 Department of Agronomy, State University of Maringá, Maringá 87020-900, Brazil; rafaromero.mendes@gmail.com (R.M.); rubem.oliveirajr@gmail.com (R.O.J.); 2 Crop and Soil Science Department, Oregon State University, Corvallis, OR 97331, USA; kazumi.s@gmail.com; 3 Embrapa Soybean, Londrina 86001-970, Brazil; fernando.adegas@embrapa.br (F.A.); francismar.marcelino@embrapa.br (F.M.-G.)

**Keywords:** Sumatran fleabane, acetolactate synthase, sulfonylureas, imidazolinones, triazolopyrimidines, resistance factor

## Abstract

Cases of weed resistant to herbicides have changed the dynamics of agricultural areas in Brazil, and in recent years, *Erigeron* species have caused major problems to farmers in the country, mainly in relation to the ineffectiveness of herbicide treatments used. The objective of this study was to confirm the cross-resistance to ALS inhibitors in populations of *Erigeron sumatrensis* as well as to investigate the existence of mutations in the site of action of ALS-inhibiting herbicides. To do this, 30 populations collected in the 2016/2017 crop season were grown in a greenhouse. Dose–response (chlorimuron-ethyl and cloransulam-methyl), inhibition of cytochrome P-450 with malathion, and ALS gene sequencing experiments were carried out in the F1 generations of two fleabane populations. The results proved the cross-resistance to chlorimuron-ethyl and cloransulam-methyl herbicides applied in the post-emergence of the resistant population of *E*. *sumatrensis*. The higher activity of P450 enzymes is unlikely responsible for the resistance of the population studied. The resistance mechanism found in R was the target site mutation Pro197Ser at the ALS gene. This is the first study in Brazil to identify a target-site change as a survival mechanism in *E*. *sumatrensis* for the resistance to ALS-inhibiting herbicides.

## 1. Introduction

Weeds known as horseweed or fleabane belong to the genus *Erigeron* (synonym *Conyza*). Three *Erigeron* species are commonly found across South America territories: *Erigeron bonariensis* (L.) Cronquist, *Erigeron sumatrensis* (L.) Cronquist, and *Erigeron canadensis* (L.) Cronquist [1]. To date, many studies in the literature report Sumatran fleabane (*Erigeron sumatrensis*) as the most frequent species infesting soybean fields in Southern Brazil [1,2,3].

Sumatran fleabane plants can reach up to 2 m, produce more than 60,000 seeds and grow exponentially for 30 days after emergence [4,5]. These species have been considered as troublesome weeds in Brazilian agricultural environments due to their potential to reduce yield in many crops. In soybeans, densities of one plant m^−2^ of hairy fleabane may result in yield losses of approximately 25%, depending on the soybean cultivar [6].

Glyphosate-resistant (GR) Sumatran fleabane was first documented in 2008 in Rio Grande do Sul [7]. Since then, several other populations have evolved resistance to glyphosate in other states such as Paraná, São Paulo, and Mato Grosso do Sul [8]. The increasing number of locations with GR *Erigeron* spp. has also increased costs of weed control, especially in burndown operations.

ALS-inhibiting herbicides are largely adopted by growers to improve the control of GR Sumatran fleabane in soybean fields. Thus, the evolution of multiple resistances (glyphosate and ALS) was rapidly reported in at least five populations from Paraná in 2012 [9]. Currently, at least 41% of all *Erigeron* populations are considered multiple resistant to both modes of action [1].

ALS is a plant enzyme that participates in the biosynthesis of branched-chain amino acids, which is critical for protein synthesis [10]. There are five chemical groups of herbicides that bind to this enzyme (i.e., sulfonylureas (SUL), imidazolinones (IMI), triazolopyrimidines (TRI), pyrimidinyl thiobenzoates (PYR), and sulfonylamino carbonyl triazolinones (SCT)). ALS inhibitors bind to the enzyme domain that crosses the entrance of its catalytic site, preventing the substrate from connecting to the enzyme and finally interrupting the synthesis of amino acids valine, leucine, and isoleucine [11].

Chlorimuron-ethyl (ethyl 2-4-chloro-6-methoxypyrimidin-2-ylcarbamoylsulfamoyl benzoate) is an ALS inhibitor from the chemical family SUL. It can be applied in pre-emergence (PRE), pre-plant incorporated (PPI), and post-emergence (POST) to control many annual broadleaf weed species. The mechanisms of the resistance of most SULs have been reported as altered herbicide-binding sites in ALS-target enzymes or metabolic deactivation [12]. The combination of chlorimuron-ethyl and glyphosate has been largely used for burndown applications prior to summer crops by Brazilian farmers. Cloransulam-methyl (methyl 3-chloro-2[[(5-ethoxy-7-fluoro[1,2,4]triazolo[1,5-c]pyrimidin-2-yl) sulfonyl]amino]benzoate is part of the chemical family TRI. As with chlorimuron-ethyl, cloransulam-methyl can also be sprayed in PRE, PPI, and POST. In soil applications, it can control important broadleaf weeds, and in combination with grass herbicides, it provides a broad control [12].

In resistant weeds, the binding affinity of ALS to the herbicide decreases as a result of target site mutations, due to amino acid substitutions in the conserved regions of the enzyme. To date, eight amino acid positions (i.e., Ala122, Pro197, Ala205, Asp376, Arg377, Trp574, Ser653, and Gly654) have been identified, causing nucleotide substitutions at the ALS gene [13]. Worldwide, cases of *Erigeron* spp. cross-resistant to ALS herbicides have been reported in Israel, the United States, and Poland [8]. In Brazil, however, although control failures of *E. sumatrensis* have been observed in fields where these herbicides are constantly used, there has been no information available so far both on the occurrence of cross-resistance among the different chemical groups of ALS herbicides and on the mechanism of resistance in those populations.

Therefore, the objective of this research was to confirm the cross-resistance to ALS-inhibiting herbicides in *E. sumatrensis* populations as well as to investigate potential mutations in the target site of the ALS gene.

## 2. Results

### 2.1. Population Screening

**Figure 1:**
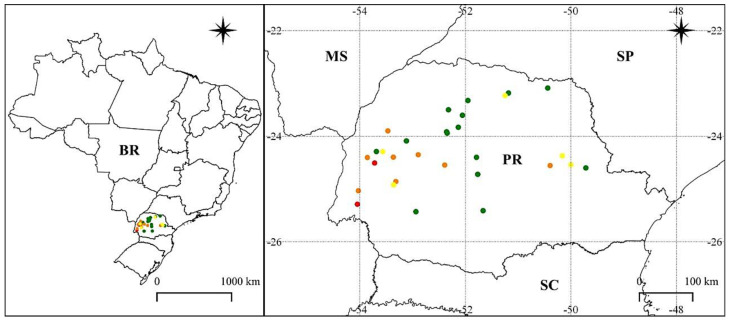
Dispersion of populations of Sumatran fleabane (*Erigeron sumatrensis*) resistant to ALS inhibitors, chlorimuron-ethyl, and cloransulam-methyl in Paraná state (PR), Brazil. Red: cross-resistance to ALS inhibitors; orange: resistant to chlorimuron-ethyl; yellow: resistant to cloransulam-methyl; green: susceptible.

From the 30 samples evaluated, only two populations (Medianeira and Maripá) presented the cross-resistance (red) to chlorimuron-ethyl and cloransulam-methyl (Figure 1).

For chlorimuron-ethyl, eight samples were classified as resistant (orange), coming from the municipalities of Campo Bonito, Juranda, Corbélia, Cascavel, Toledo, Nova Aurora, Tibagi, and Roncador. Regarding cloransulam-methyl, five populations were classified as resistant (yellow). These populations were identified in the municipalities of Campina da Lagoa, Cambé, Assis Chateaubriand, Ventania, and Piraí do Sul.

The remaining populations were classified as susceptible, since their injury exceeded 80% for both ALS inhibitors, and were represented in the green color on the map (Figure 1). For the subsequent study, the population collected in Maripá was considered resistant (R), and the population collected in Engenheiro Beltrão was classified as susceptible (S).

### 2.2. Whole-Plant Dose–Response Results

**Figure 2:**
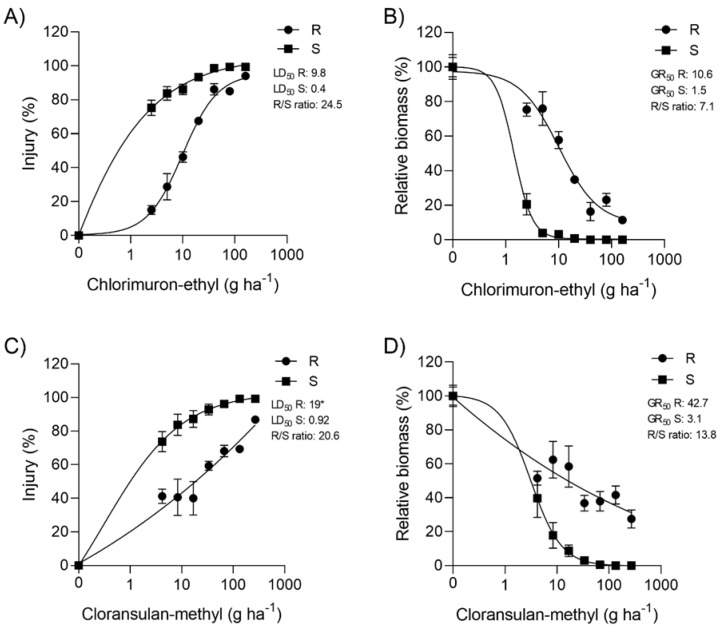
Dose-response curves for chlorimuron-ethyl (**A**,**B**) and cloransulam-methyl (**C**,**D**) in populations resistant (R) and susceptible (S) to ALS inhibitors (*Erigeron sumatrensis*).

With growth reduction by 50% (GR_50_) values of 1.5 g ha^−1^ for chlorimuron-ethyl and 3.1 g ha^−1^ for cloransulam-methyl, the S population demonstrated high sensitivity to both herbicides. For R, the GR_50_ values were 10.6 and 42.7 for chlorimuron-ethyl and cloransulam-methyl, respectively, generating resistance factors (RFs) of 7.1 (chlorimuron-ethyl) and 13.8 (cloransulam-methyl) (Figure 2B,D). The results based on the injury data (Figure 2A,C) resulted in RF values of 24.5 and 20.6 for chlorimuron-ethyl and cloransulam-methyl, respectively. The results indicated the cross-resistance to SUL and TRI ALS groups in R.

### 2.3. Screening for ALS Inhibitors

In both experiments, there was a significant interaction of herbicide treatments with the populations. In the PRE experiment, the S was sensitive to the range of doses applied, demonstrating injury levels higher than 99% (Table 1), which was expected responses to herbicides applied in the PRE of susceptible plants. However, for R, none of the herbicides applied were effective to achieve satisfactory control levels (≥90%).

The limited activity of PRE-treatments in population R was observed, due to not only the lack of prevention of seedlings emergence, but also no severe injuries to them at 28 days after application (DAA).

In the POST, for the S population, the herbicides from the chemical groups SUL and TRI provided injuries of ≥90%, with no differences among them. Regarding the chemical groups IMI and PYR, the lowest injury was observed for pyrithiobac-sodium (51.2%), followed by those for imazethapyr (68.7%) and imazapic (83.7%) (Table 1).

For the R population, acceptable control levels (≥90%) were not found in any of the tested herbicides. The highest injury levels were observed for imazapic (75%), followed by those for chlorimuron-ethyl (66.2%), trifloxysulfuron-sodium (65%), and cloransulam-methyl (61.2%). Herbicide pyrithiobac-sodium caused only 5% of injury to this population.

### 2.4. Inhibition of Cytochrome P-450 with Malathion

For the R population, as well as for the S population, no differences were observed between the application of isolated herbicides and associations with malathion (Table 2).

### 2.5. ALS Sequencing

**Figure 3:**
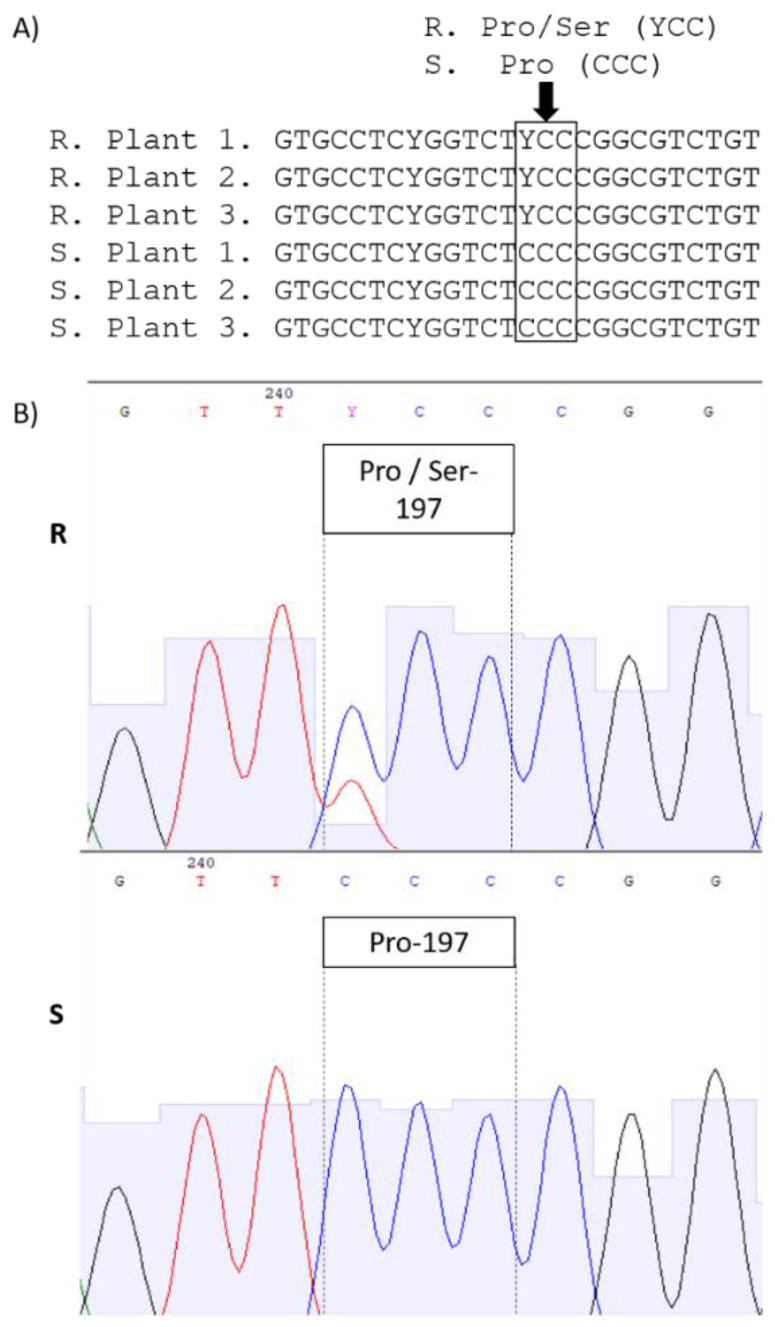
Nucleotide sequence of the ALS gene of *Erigeron sumatrensis* resistant (R) and susceptible (S) to ALS inhibitors. (**A**) Substitution of Ser for Pro in position 197. Letter Y represents base T or C. (**B**) Chromatogram demonstrating the heterozygosity found in the substitution of alleles containing CCC (plants S) and TCC or CCC plants R.

By aligning and comparing the ALS gene sequences of the S and R plants, an amino acid substitution was observed at 197 residue of the gene (based on the sequence of *Arabidopsis thaliana*). All sequences of the R had a Ser, instead of a Pro, in this position (Pro197Ser), due to the substitution of CCC nucleotide by TCC (Figure 3). However, the R plants were heterozygous for this mutation, because overlapping peaks were observed in the chromatograms (Figure 3B). The sequences have been deposited in the National Center for Biotechnology Information (NCBI; accession numbers: MZ816699, MZ816700, and MZ816701).

## 3. Discussion

In Brazil, a previous research found populations of *E. sumatrensis* highly resistant to chlorimuron-ethyl [9]. However, studies confirming cross-resistance to cloransulam-methyl in *Erigeron* spp. have never been published so far.

In *Erigeron* species, ALS inhibitors were chronologically the second mode of action that had efficiency compromised by the selection of resistant biotypes in mid-2014 [9]. Since then, several studies have reported multiple resistance in some populations, such as a biotype resistant to EPSPS inhibitors, ALS inhibitors, and photosystem I inhibitors [3]. Another case was described with the resistance to these same mechanisms plus photosystem II inhibitory and protophorphyrinogen-oxidase (PPO) inhibitors [2]. The population evaluated here (R-Maripá) was submitted to the application of all these herbicides in preliminary work, and except for EPSPS inhibitors, the possibility of resistance to other mechanisms of action was discarded (data not shown).

Although several *Erigeron* populations have been selected for resistance to ALS inhibitors, mainly in Paraná [1], the resistance mechanism has not yet been investigated. It is worth mentioning that different patterns of cross-resistance may occur, depending on the selection pressure imposed at each location, and this may result in the evolution of different resistance mechanisms [1]. In addition, the absence of patterns among populations of *Erigeron* spp. may be linked to the innate characteristics of the genus, with a high genetic divergence between the three species of *Erigeron* and the genetic diversity between populations of the same species, as observed in *E. sumatrensis* by researchers [14].

High levels of resistance to ALS inhibitors are commonly found in weeds. In *E. canadensis* populations from Israel, the resistances to three chemical groups (imidazolinones, sulfonylureas, and pyrimidinyl-thio-benzoates) were reported due to the substitution of Trp574 for Leu. The RFs of >29, >42, and >44 were found for imidazolinones, sulfonylureas, and pyrimidinyl-thio-benzoates, respectively [15].

Researchers evaluated cross-resistance patterns in three *E. canadensis* populations to four ALS-inhibiting herbicides from different chemical groups. Populations P525 and P13 showed different patterns of resistance when compared to population P116, since they were susceptible to imazethapyr (RF GR_50_ = 0.9 and 0.1, respectively), but resistant to herbicides from other chemical groups. On the other hand, population P116 was cross-resistance to all four herbicides evaluated, one from each chemical group (RF GR_50_: 33 (cloransulam-methyl), 34 (chlorimuron-ethyl), 9.1 (imazethapyr), and 580 (bispyribac)) [16].

In our study, the herbicides studied did not prevent the emergence of seedlings. These results corroborate those found by Guerra et al. who found that all chemical groups tested provided unsatisfactory control when evaluating dose-response relationships in PRE for ALS inhibiting herbicides (imazethapyr, chlorimuron-ethyl, diclosulam, imazaquin, and, flumetsulam) in a *Bidens pilosa*-resistant biotype, even using the two times recommended dose [17].

In samples of *Amaranthus retroflexus* resistant to trifloxysulfuron-sodium and pyrithiobac-sodium from cotton producing regions in Brazil, high resistance factors were observed both in POST and PRE [18]. With inhibitors of carotenoid synthesis, the resistance to herbicide mesotrione was confirmed in PRE in *Amaranthus. tuberculatus* biotypes, in which the resistance in POST applications of this product has already been observed [19].

For the last two decades, glyphosate and chlorimuron-ethyl have been frequently used as the basis for burndown applications prior to summer crops in many areas of grain production. After the selection of GR *Erigeron* spp., many farmers have tried alternative herbicides, usually ALS inhibitors, to mitigate limited control results [20,21]. This may have contributed to the selection of this R population with resistance to diclosulam, chlorimuron-ethyl, metsulfuron-methyl, and imazethapyr in the PRE.

Resistance due to enhanced metabolism resistance was studied in an indirect basis, using a cytochrome P450 inhibitor, malathion. The application of malathion in association with chloransulam-methyl, chlorimuron-ethyl, or imazethapyr does not annul the resistance in the populations studied. This type of effect was also seen in *Raphanus sativus* plants resistant to iodosulfuron or imazethapyr in Southern Brazil, for which the addition of malathion does not influence the injury levels of resistant populations. In this case, the mechanism responsible for resistance is the Trp-574 mutation [22].

Cytochromes P450 monooxygenases catalyze a range of hydroxylation and oxidation reactions that can occur with a given herbicide, and in some cases, this process may be responsible for resistance to a given herbicide [23].

However, in the R population of *E. sumatrensis*, there was no synergism between the tested ALS-inhibiting herbicides and the organophosphate malathion, which indicates that ALS inhibitors metabolism by P450 enzymes is not the mechanism involved in the resistance of this population. Malathion may not inhibit all P450 enzymes, and then, further investigations are necessary since metabolism can simultaneously contribute with other mechanisms of ALS-resistant plants [24].

In sequencing the ALS gene, the substitution of Pro197Ser was observed in the R plants, with heterozygosity. As fleabane species are hexaploid [25], heterozygous mutations are commonly found. However, it is not possible to clarify in this work what is the frequency of the Pro197Ser among all ALS alleles. Heterozygosis in mutations that provide resistance to herbicides in polyploid species are frequently reported, for example in GR *Bidens subalternans*, *Euphorbia heterophylla* [26,27], and *Lolium multiflorum* resistant to inhibitors of ALS [28].

The Pro197Ser mutation is the most common of all ever reported in the ALS gene sequence [13]. In other weed species, this substitution normally causes high levels of resistance to SUL and certain susceptibility (low resistant factors) to the other chemical groups of ALS inhibitors [13]. In a species belonging to the same genus (*E. canadensis*), Pro197Ser was found to provide high levels of resistance to chlorimuron-ethyl and cloransulam-methyl [16], which is consistent with what was observed in the present study.

Some mutations already described in the literature confer cross-resistance patterns that are similar to those found in this work for *E. sumatrensis*. Matzrafi et al. reported the occurrence of cross-resistance in populations of *E. canadensis* to the group of IMI, SUL, and PYR due to the target site mutation Trp574 by Leu [15]. For the same species, the substitution Pro197Ser confers resistance to all chemical groups that inhibit ALS, except for IMI [16]. As for other species, different patterns of cross-resistance can be found. For example, when Pro197 is substituted by Leu instead of Ser, resistance is likely to manifest in relation to all groups (IMI, SUL, and TRI), as already described for *Amaranthus palmeri* [29]. Therefore, the Pro197Ser mutation may not be the only mechanism involved in *E. sumatrensis* in Brazil, which leads to the need for investigating more resistant populations in future studies. However, in the present study, the only mutation found was Pro197Ser.

Species of the *Erigeron* complex have become the most challenging weed problem in South American territories due to the cases of multiple resistances and the consequent high investment for control [30]. Before investigating control alternatives for these populations, it is essential to understand the resistance mechanisms for each herbicide and thus propose specific management strategies. In addition, weed management practices for mitigating resistance evolution in these species should be widely adopted, including crop rotation, straw input in the soil, mode of actions rotation, and prevention of seed entry into properties [31].

## 4. Materials and Methods

### 4.1. Population Screening

*E. sumatrensis* seeds were collected at a physiological maturation stage, with samples made up of 5 to 10 plants per location. The seeds were stored in paper bags, identified and stored at room temperature until the beginning of the experiments. The collection sites were agricultural properties where soybean was cultivated, and failures in the control of *E. sumatrensis* were observed after the application of glyphosate and chlorimuron-ethyl herbicides in the 2016/2017 season. A total of 30 populations were collected in the state of Paraná in February 2017 (Table 3).

In June 2017, the seeds were sown in trays containing a substrate Carolina Soil^®^ (CSC, Brazil) which was 0.5 cm deep, and after seedling emergence, they were transplanted into 0.2 L pots. For each population, four repetitions were used. These populations received a POST application of chlorimuron-ethyl (Classic^®^; 20 g a.i. ha^−1^) and cloransulam-methyl (Pacto^®^; 33.6 g a.i. ha^−1^), both applied with mineral oil (0.5% *v v*^−1^) at the 5–7 leaf stages.

The visual injury percentages were evaluated at 28 DAA using the 0–100% scale with 0% meaning the absence of symptoms and 100% indicating the death of the plant. The populations considered resistant were those in which plants survived herbicidal treatments (below 80% injury) in at least three of the four replications [32].

Experiments were conducted in a greenhouse under natural light and temperature conditions with plenty of water. Herbicides were sprayed using a CO_2_-pressurized backpack sprayer equipped with three flat-fan nozzles (XR110015, TeeJet Technologies), which delivered 150 L ha^−1^. These conditions were repeated throughout the study.

### 4.2. Whole-Plant Dose-Response Relationship

Dose-response experiments were conducted in resistant (R) populations that survived with treatments with both chlorimuron-ethyl and cloransulam-methyl in the screening. Seeds from the R population collected in Maripá (#17) and from a known susceptible population (S) collected in Engenheiro Beltrão (#1) were sown in trays and transplanted into 1 L pots under the same conditions as the screening experiments.

The experiments were conducted in a 2 × 8 factorial design, in which the first factor was constituted by the two populations of *E. sumatrensis* (R and S) and the second factor consisted of 8 herbicides doses. The doses were proportionally arranged: 0, 1/8, 1/4, ½, 1, 2, 4, and 8 times the recommended dose of each herbicide. For chlorimuron-ethyl, the recommended dose adopted was 20 g a.i. ha^−1^, and for cloransulam-methyl, the recommended dose adopted was 33.6 g a.i. ha^−1^. Each herbicide constituted an independent experiment. At 28 DAA, visual injury was evaluated (scale: 0–100%), and plant shoots were collected and dried in an oven at 65 °C for four days for dry mass measurement. The experiments were conducted in a completely randomized design with four replications, and the experiments were repeated over time.

### 4.3. Screening for the Cross-Resistance for ALS Inhibitors

Two experiments were conducted in a completely randomized design, with four replications. The first experiment was carried out in a 5 × 2 factorial design, and the first factor corresponded to four herbicidal treatments applied in PRE and a non-treated control (Table 4). The second factor was constituted by the two populations (R and S) of *E. sumatrensis*.

Two hundred seeds of R and S populations were sown (0.5 cm deep) in each experimental unit, which received 10 mm of irrigation right after sowing. The applications of the treatments in PRE occurred right after irrigation.

The second experiment was carried out in an 8 × 2 factorial design. The first factor corresponded to seven herbicidal treatments applied in POST, plus a non-treated control (Table 4). The second factor was constituted by the same populations (R and S) of *E. sumatrensis*, and plants had 5 to 7 leaves at the moment of the herbicide application. The sowing and transplantation method was the same used until then for the experiments described above. Not all herbicides used were registered for use in *Erigeron* spp.; however, the dose used was based on the recommendations for other broadleaf species, both for the PRE and the POST [33]. Both experiments were conducted in a completely randomized design with four replications and were repeated over time.

### 4.4. Inhibition of Cytochrome P-450 with Malathion

Seeds of the F1 generation of the R and S populations were sown in trays and transplanted into pots exactly as performed in the previous stages of this work.

Two experiments were carried out, one for each population (R and S). The experiments consisted of the application of cloransulam-methyl (33.2 g ha^−1^), chlorimuron-ethyl (20 g ha^−1^), and imazethapyr (106 g ha^−1^) with or without the application of malathion (1000 g ha^−1^) one hour before herbicide treatment. The experimental design used was completely randomized, with four replications. At 28 DAA, visual injury (scale: 0–100%) and shoots dry mass were evaluated.

### 4.5. DNA Extraction, Amplification, and ALS Gene Sequencing

Approximately 100 mg of fresh leaf tissue from the plants of the R and S populations were collected and frozen in liquid nitrogen. Three R and three S plants had their DNA extracted, and the tissue was grinded in test tubes with metal balls in a mechanical grinder. The DNA was extracted using the CTAB detergent method, as described by [34]. The samples were quantified in Nanodrop^®^ (Thermo Fisher Scientific, Waltham, MA, USA) with agarose gel (1%) to verify the quality of the extraction. The primer pairs to amplify the ALS gene from fleabane were the same used by [16]. Three pairs of primers covering the entire ALS sequence were used, overlapping the amplified sequences from each other at the end of each fragment. All regions known to have mutations in plants resistant to ALS inhibitors were amplified, being Ala122, Pro197, Ala205, Asp376, Arg377, Trp574, and Ser653. PCR reactions were performed by adding 20 ng of DNA, 1 μL (10 μM) of each primer, 2 μL of 10× Taq Buffer, 0.75 MgCl2 (50 μM), 1.5 μL of DNTPs, 0.2 μL of Platinum^®^ *Taq* DNA polymerase High Fidelity (Life Technologies, United States of America), and nuclease-free water, until the mixture reached a final volume of 20 μL. PCR was conducted according to the following schedule: denaturation at 94 °C for 3 min, 40 cycles of 94 °C for 45 s, 53 °C for 45 s, and 72 °C for 1 min 30 s, followed by a final extension of 10 min at 72 °C. The PCR product was purified by cutting the bands of the agarose gel (2%) using the Wizard^®^ SV gel and PCR Clean-up System (Promega, Brazil) and sequenced in Sanger sequencing (M13 Universal Nucleotides). Bioedit software was used to align the gene fragments and the sequences of three plants from the R populations and three plants from the S populations.

### 4.6. Data Analysis

Data from dose-response experiments were subjected to the nonlinear regression adjustment of three parameters [35], described as Equation (1):

(1) $$Y=\frac{a}{\left[1-{\left(\frac{x}{I50}\right)}^{b}\right]},$$

where *Y* is the injury or biomass (relative to non-treated) at 28 DAA (dependent variable); *x* corresponds to the dose of the herbicide (g ha^−1^) (independent variable); *a* is the asymptote between the maximum and minimum points of the variable; *I*_50_ is the dose that provides 50% of the asymptote, which can be for injury (LD_50_) or relative biomass (GR_50_); and *b* represents the slope of the curve around *I*_50_ (inflection point of the curve).

For the screening experiment for the cross-resistance and application of P450 inhibitor, the data were subjected to the analysis of variance and subsequent comparison of means by the Student *t*-test (5%).

## 5. Conclusions

The population of *E. sumatrensis* investigated in the study had the cross-resistance to ALS inhibitors chlorimuron-ethyl and cloransulam-methyl. Malathion spraying before ALS-inhibitors applications did not revert the resistance, indicating that the higher activity of P450 enzymes in R plants is not the mechanism of resistance involved. The heterozygous mutation Pro197Ser found at the ALS gene was the mechanism that provided resistance in plants of this population. This study found and summarized a target-site mutation clarifying the resistance mechanism in ALS-resistant Sumatran fleabane for the first time in Brazil.

## Figures and Tables

**Table 1:** Percentages of injury at 28 days after application (DAA) of two *Erigeron sumatrensis* populations after the application of ALS-inhibiting herbicides in Pre- and Post-emergence.

Herbicide (Dose in g ha^−1^)	Pre-Emergence Injury (%)			
	R		S	
Non-treated	0	c	0	b
Diclosulam (25.2)	65	a	100	a
Chlorimuron-ethyl (20)	31.2	b	100	a
Metsulfuron-methyl (2)	71.2	a	99	a
Imazethapyr (106)	38.7	b	100	a
**Herbicide**	**Post-Emergence Injury (%)**			
	**R**		**S**	
Non-treated	0	e	0	e
Cloransulam-methyl (33.6)	61.2	bc	99.5	a
Chlorimuron-ethyl (20)	66.2	ab	95.5	a
Metsulfuron-methyl (2)	51.2	d	98.2	a
Trifloxysulfuron-sodium (7.5)	65	b	90	ab
Imazethapyr (106)	52.5	cd	68.7	c
Imazapic (98)	75	a	83.7	b
Pyrithiobac-sodium (36)	5	e	51.2	d

Means followed by the same lower-case letter in each experiment and in the same column do not differ by the Student’s *t*-test at a 5% probability. Pre-emergence coefficient of variation percentage (CV %) = 24.52; Post-emergence CV (%) = 11.51.

**Table 2:** Percentages of injury and dry masses at 28 DAA of two *Erigeron sumatrensis* populations after the application of ALS-inhibiting herbicides with or without the application of malathion.

Herbicide (Dose in g ha^−1^)	R				S			
	Injury (%)		Dry Mass *		Injury (%)		Dry Mass *	
Non-treated	0	f	100	a	0	c	100	a
Malathion (1000)	0	f	84.5	b	0	c	108.5	a
Cloransulam-methyl (33.6)	22.5	e	73.8	bc	99.5	a	0	c
Cloransulam-methyl (33.6) ^+^	37.5	d	58.8	cd	99.5	a	0	c
Chlorimuron-ethyl (20)	47.5	bc	69.9	bc	99.5	a	0	c
Chlorimuron-ethyl (20) ^+^	42.5	cd	60.1	c	98.2	a	0	c
Imazethapyr (106)	48.7	b	53.4	de	62.5	b	41.8	b
Imazethapyr (106)^+^	56.2	a	42.3	e	61.2	b	33.9	b

^+^ with the application of malathion (1000 g ha^–1^) one hour before herbicide treatment; * % untreated. Means followed by the same lower-case letter in the column do not differ by the Student’s *t*-test at a 5% probability. Injury R population CV (%) = 13.39; dry mass R population CV (%) = 15.65; injury S population CV (%) = 2.62; dry mass S population CV (%) = 23.69.

**Table 3:** Identification of populations and sampling sites for *Erigeron sumatrensis* seeds in Maringá, PR, 2017.

Population Code	City	State	Geographical Coordinates	
			Latitude	Longitude
1	Engenheiro Beltrão	PR	23°50′23″ S	52°22′49″ W
2	Campina da Lagoa	PR	24°55′17″ S	53°21′35″ W
3	Medianeira	PR	25°17′22″ S	54°02′50″ W
4	Campo Bonito	PR	25°02′02″ S	54°01′42″ W
5	Quedas do Iguaçu	PR	25°25′48″ S	52°56′15″ W
6	Peabiru	PR	23°56′31″ S	52°20′43″ W
7	Campo Mourão	PR	23°53′35″ S	52°21′38″ W
8	Juranda	PR	24°21′07″ S	52°53′38″ W
9	Palotina	PR	24°17′26″ S	53°41′12″ W
10	Corbélia	PR	24°51′32″ S	53°19′4″ W
11	Cascavel	PR	23°53′59″ S	53°28′9″ W
12	Floresta	PR	23°36′14″ S	52°3′21″ W
13	Maringá	PR	23°19′26″ S	51°57′6″ W
14	Bandeirantes	PR	123°5′16″ S	50°26′20″ W
15	São Jorge do Ivaí	PR	23°29′57″ S	52°19′14″ W
16	Goioerê	PR	24°05′23″ S	53°07′01″ W
17	Maripá	PR	24°30′25″ S	53°43′12” W
18	Toledo	PR	24°24′14″ S	53°51′29” W
19	Londrina	PR	23°10′56″ S	51°10′57” W
20	Cambé	PR	23°13′52″ S	51°14′47″ W
21	Nova Aurora	PR	24°23′51″ S	53°21′55” W
22	Assis Chateaubriand	PR	24°17′34″ S	53°33′56″ W
23	Ventania	PR	24°22′18″ S	50°9′42″ W
24	Piraí do Sul	PR	24°32′28″ S	49°59′56″ W
25	Ivaiporã	PR	24°24′2″ S	51°47′23″ W
26	Pitanga	PR	24°43′22″ S	51°45′57″ W
27	Tibagi	PR	24°33′24″ S	50°23′37″ W
28	Castro	PR	24°36′4″ S	49°42′58″ W
29	Guarapuava	PR	25°24′42″ S	51°39′52″ W
30	Roncador	PR	24°32′49″ S	52°23′29″ W

**Table 4:** Herbicides, label doses used in Brazil (g a.i. or a.e. ha^−1^), and application modality for cross-resistance experiments.

Treatments Exp 1 (Pre-Emergence)	Chemical Group	Treatments Exp 2 (Post-Emergence)	Chemical Group
Non-treated	-	Non treated	-
Diclosulam (25.2)	TRI	Cloransulam-methyl ^1/^ (33.6)	TRI
Chlorimuron-ethyl (20)	SUL	Chlorimuron-ethyl ^2/^ (20)	SUL
Metsulfuron-methyl (2)	SUL	Metsulfuron-methyl ^2/^ (2)	SUL
Imazethapyr (106)	IMI	Trifloxysulfuron-sodium (7.5)	SUL
		Imazethapyr (106)	IMI
		Imazapic (98)	IMI
		Pyrithiobac-sodium (36)	PYR

SUL, sulfonylureas; IMI, imidazolinones; TRI, triazolopyrimidines; PYR, pyrimidinyl thiobenzoates. ^1/^ Applied with a non-ionic surfactant (0.2% *v v*^−1^); ^2/^ applied with mineral oil (0.5% *v v*^−1^).

## Data Availability

Not applicable.
